# A high-power and fast charging Li-ion battery with outstanding cycle-life

**DOI:** 10.1038/s41598-017-01236-y

**Published:** 2017-04-24

**Authors:** M. Agostini, S. Brutti, M. A. Navarra, S. Panero, P. Reale, A. Matic, B. Scrosati

**Affiliations:** 1 Department of Applied Physics, Chalmers University of Technology, SE-41296 Göteborg, Sweden; 2 CNR-ISC, U.O.S. Sapienza, Piazzale A. Moro 5, 00185 Roma, Italy; 3 Dipartimento di Scienze, Università della Basilicata, V.le Ateneo Lucano 10, 85100 Potenza, Italy; 4 Dipartimento di Chimica, Sapienza Università di Roma, P.le Aldo Moro 5, 00185 Roma, Italy; 5 ENEA-Centro di Ricerca Casaccia, Via Anguillarese, 00100 Roma, Italy; 6 Helmholtz-Institut Ulm (HIU), Ulm, Germany

## Abstract

Electrochemical energy storage devices based on Li-ion cells currently power almost all electronic devices and power tools. The development of new Li-ion cell configurations by incorporating innovative functional components (electrode materials and electrolyte formulations) will allow to bring this technology beyond mobile electronics and to boost performance largely beyond the state-of-the-art. Here we demonstrate a new full Li-ion cell constituted by a high-potential cathode material, i.e. LiNi_0.5_Mn_1.5_O_4_, a safe nanostructured anode material, i.e. TiO_2_, and a composite electrolyte made by a mixture of an ionic liquid suitable for high potential applications, i.e. Pyr_1,4_PF_6_, a lithium salt, i.e. LiPF_6_, and standard organic carbonates. The final cell configuration is able to reversibly cycle lithium for thousands of cycles at 1000 mAg^−1^ and a capacity retention of 65% at cycle 2000.

## Introduction

Energy conversion and storage are key enabling technologies that will pave the way in the XXI century to mass electro-mobility, smart-grids of continental-size and realistic reduction of CO_2_ emissions. Electrochemical energy storage devices based on Li-ion cells currently power almost all electronic devices. Breakthrough progresses in Li-ion batteries (LIBs) can be achieved in terms of higher power performance, longer cycle life, improved safety and sustainability^1^ by the development of anodes, cathodes and electrolytes materials relying on innovative chemistries^2, 3^.

Here we propose and demonstrate a novel formulation of a full lithium ion cell. The key-innovation stands in the unique combination of (a) a nanostructure TiO_2_-based negative electrode with a tailored 1-D tubular morphology; (b) a LiNi_0.5_Mn_1.5_O_4_-based positive electrode (LNMO) with a finely tuned stoichiometry and a surface layer obtained through a single-stage, simple, cheap and easy-scalable mechanochemical milling route followed by high temperature annealing in air; and (c) a composite liquid electrolyte formed by a mixture of LiPF_6_, ethylene carbonate, dimethyl carbonate and N-n-butyl-N-methylpyrrolidinium hexafluorophosphate (Py_14_PF_6_) ionic liquid with optimized composition^4^. This full cell configuration is able to provide outstanding performance in terms of power density and cycling life, in combination with an intrinsically higher safety, compared to commercial cells, provided by the ionic liquid component, and lower costs as well as an improved environmental compatibility due to the absence of cobalt in the cathode material.

In the current literature, a huge number of possible alternative configurations for next generation lithium-ion cells have been proposed, based on a variety of different chemistries at the cathode and anode sides and for the electrolyte^5–7^. Among them, the concept of a 3–3.5 V Li-ion cell made by coupling LNMO spinel and TiO_2_-based anodes has been demonstrated^8, 9^.

Titanium oxide-based anodes have relevant advantages compared to graphite and conversion/alloying materials: (a) the working potential falls within the thermodynamic stability window of the standard organic carbonate electrolytes (>0.8 V vs. Li); (b) titanium oxide-based materials can be easily obtained as nano-particulates by tuning the synthetic conditions, thus disclosing excellent power performance^10^; their density is two times larger than graphite and therefore the volumetric performance can double compared to a standard graphite-based Li-ion cells^10^. Unfortunately, their high operating potential (1.5 V vs Li) is also an important drawback for the full cell energy density. Thus, they need to be coupled with high-potential cathodes, e.g. LNMO or others like LiCoPO_4_^3^, to achieve competitive performance with respect to the state-of-the-art formulations^1^.

Turning to the cathode side, the high voltage LNMO spinel oxide, is one of the most promising cathode materials due to the large reversible capacity, high thermal stability, low cost and null content of the toxic, high cost and pollutant cobalt^11^. The key-point to achieve excellent power performance from this material is the optimization of the synthetic procedure to obtain well-formed particles with optimal morphology^11^. However, the adoption of a simple and single-step synthesis strategy to optimize the crystallinity, composition, morphology and surface properties to be able to fully address the serious capacity fading of LNMO cathodes, especially at high rate and at elevated temperatures, has never been reported^3^. In fact, only the combination of a suitable lattice doping with coating layers through complex and expensive multi-stage synthetic procedures is apparently able to lead to materials with superior properties in lithium cells^12^.

The main reason of the capacity fading of the LNMO electrodes upon cycling roots is in the complex parasitic chemistry that takes place at high potentials onto the positive electrode surface^13–15^. It is a matter of fact that the adoption of any high potential positive electrode materials, in combination with commercial carbonate-based electrolytes, results in a massive increase of parasitic reactivity upon cycling above 4.2–4.5 V vs Li^16, 17^. This unavoidable effect impacts negatively the long-cycling performance and self-discharge, leading to rapid battery failure. Additives and use of non-carbonate based co-solvents have been proposed in the literature^16, 18, 19^ but, so far, no ultimate solution for stable liquid electrolytes above 4.2–4.5 V vs. Li has been found^13^.

To address the shortcomings at high potentials outlined above and to improve the safety of the battery we developed a composite solution, made by mixing an ionic liquid (IL) component, Py_14_PF_6_, with a conventional LiPF_6_-alkyl carbonate based electrolyte (i.e. the commercial LP30 SelectiLyte™) to obtain an innovative electrolyte able to operate at high potentials and with improved thermal stability. The LiPF_6_ salt has a unique set of properties for its successful use in lithium battery electrolytes, including the ability to achieve high ionic conductivity and negligible reactivity towards aluminum current collectors. Even though widely adopted, the use of LiPF_6_ combined with alkyl carbonate solvents imposes some limitations. The main issues are the safety hazards related to the volatility of carbonate-components and the limited temperature range for safe and practical ion conduction. Hybridization of volatile carbonates with ionic liquid components was proved to be effective in reducing flammability of the electrolyte^20, 21^. The IL-LP30 composition selected in this work was already introduced by us in ref. 4, where we demonstrated: (i) the suppression of IL crystallization and smoothing of the melting/crystallization features of LP30 component, with a shift to lower temperatures, thus extending applicability of the composite electrolyte down to −30 °C; (ii) an impressive ionic conductivity, both at room temperature (≥10^−2^ S cm^−1^) and at sub-zero (>10^−3^ S cm^−1^ at −30 °C); (iii) an improvement of the anodic stability of the hybrid electrolyte compared to bare LP30, with well-controlled decomposition current densities lower than 0.1 mA cm^−2^ up to 5.1 V vs Li. It is also found that this new composite electrolyte has a flash point increase of 10 °C with respect to the commercial LP30 solution (i.e. 32 °C vs. 22 °C). Thus, the coupling of this innovative electrolyte with a high potential positive electrode material, i.e. LNMO, and an intrinsically safe negative electrode material, i.e. TiO_2_, allows a final cell configuration with intrinsic chemical safety comparable with the safest carbonate-based Li-ion cell on market, i.e. the LiFePO_4_/Li_4_Ti_5_O_12_ (LFP-LTO) one, but with a mean working voltage of about 2.7–3 V, well above the 1.9 V of the LFP-LTO cell.

In summary, the here-proposed full lithium ion cell formulation exploits three simultaneous innovations, on the two electrodes sides as well as for the electrolyte, to disclose outstanding and unpreceded power performance and cycling life compared to the state-of-the-art, with a parallel improvement in the intrinsic safety of the device through the use of an ionic liquid component and the full environmental benignity by replacing cobalt in the cathode active material.

## Results

**Figure 1:**
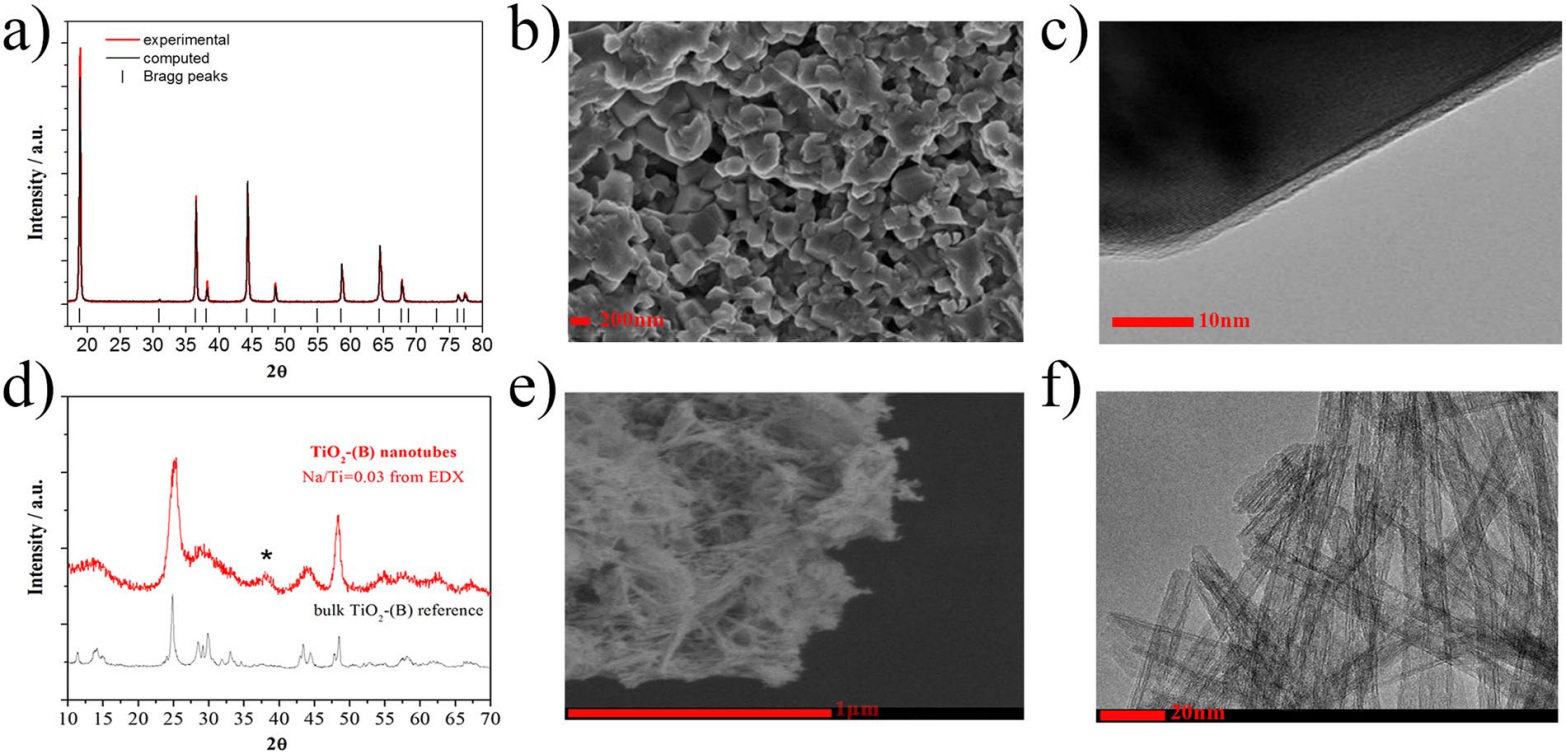
(**a**) XRD pattern, (**b**) SEM and (**c**) TEM micrographies of the LNMO powder. (**d**) XRD pattern, (**e**) SEM and (**f**) TEM micrographies of the TiO_2_-B powder.

LNMO is an iron and chromium doped spinel with the exact stoichiometry Li_0.98_Ni_0.51_Mn_1.39_Fe_0.11_Cr_0.01_O_4±δ_. The X-ray diffraction pattern is shown in Fig. 1a and the Rietveld refinement performed on the pattern reveals a poorly ordered lattice, where transition metals randomly occupy the octahedral 16d sites in a Fd-3m symmetry (space group 227), with a rather small lattice parameter, i.e. 8.178 ± 0.005 Å, but extended crystallite domains, i.e. 124 nm. Figure 1b and c report the morphological characteristics of the LNMO as emerging from SEM and TEM analysis, respectively.

Figure 1b shows a high homogeneity of the LNMO particles with an average size of about 200 nm. The image shows well-formed prismatic crystallites with sharp edges and regular shape. Figure 1c shows the detail of a particle, demonstrating how the particle is well crystallized in the bulk, while the surface is homogeneously covered by an amorphous 1-to-2 nanometer thick layer. Preliminary X-ray Photoelectron Spectroscopy (XPS) data suggest that this native surface layer is mainly constituted by Cr(III) oxide and spontaneously grows on the surface of well-formed crystalline iron-doped LNMO particles at 800 °C. A more detailed description will be discussed elsewhere.

**Figure 2:**
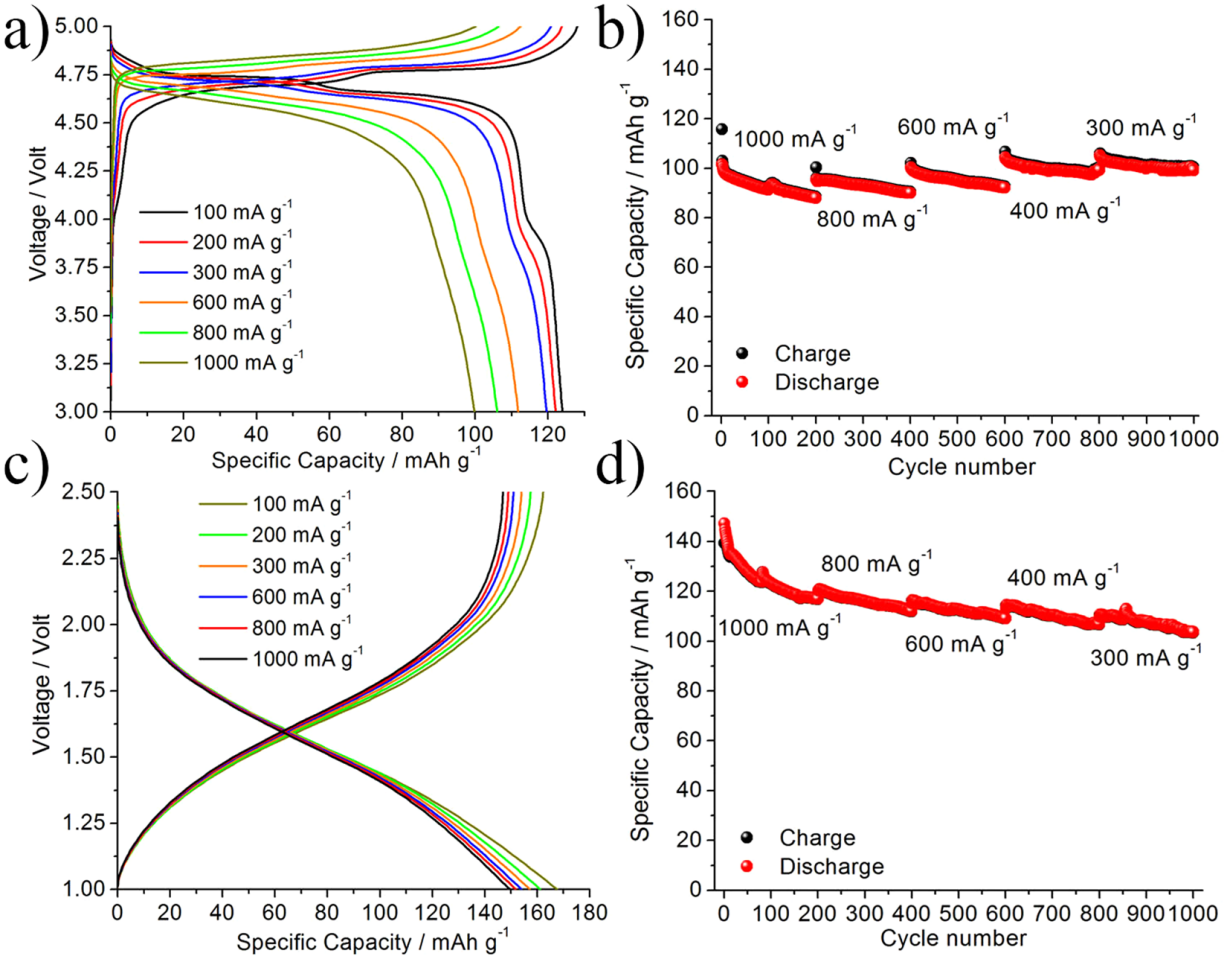
(**a**) Voltage profiles and (**b**) specific capacity performance of the Li/LP30/LNMO half-cell at different current rates. (**c**) Voltage profiles and (**d**) specific capacity performance of the Li/LP30/TiO_2_-B half-cell at different current rates.

The X-ray diffraction pattern of the anode material corresponds to monoclinic TiO_2_-B, see Fig. 1d ^22^. The peak broadening results from the nanoscale of the sample which is also seen in the SEM image, Fig. 1e, where the TiO_2_-nanotubes highlight a needles-like network mainly elongated along the *b* axis for several microns. Moreover, the TEM image in Fig. 1f reveals a tube diameter of about 10 nm and a wall thickness of about 2.5 ÷ 3 nm. The electrochemical performance of the LNMO and TiO_2_-nanotube materials have first been investigated in combination with a standard LP30 electrolyte solution by using half-lithium cells configuration (see Methods section). Electrochemical results are summarized in Fig. 2, where the voltage profiles (a,c) and the specific capacity vs cycle number (b,d) at different current rates of the LNMO and of the TiO_2_-nanotubes, are reported. The LNMO electrode shows low overpotentials (see Fig. 2a) and a minor capacity decrease even at very high current rates. LNMO is able to exchange approximately 125 mAh g^−1^ at 100 mA g^−1^ and 100 mAh g^−1^ at current as high as 1000 mA g^−1^. The charge-discharge voltage hysteresis increases from 30 mV to 200 mV by increasing the applied current 10 times from 100 to 1000 mA g^−1^. It is worth noting that at the lower current rates a limited contribution of Mn^3+^/Mn^4+^ redox process^23^ is distinguishable around 4 V vs Li, that disappears as the current rate increases. Upon cycling, the LNMO electrode shows a remarkably stable performance even after 1000 cycles of electrochemical lithium de-insertion/insertion (see Fig. 2b), performed at different current-rates, i.e. from an initial value of 10C to the final value of 3C. This outstanding rate performance confirms the synergetic effect of the co-doping of the LNMO spinel lattice by Cr(III) and Fe(III)^24^. On the other hand, the prolonged cycling reversibility may be related to the uniform Cr_2_O_3_ amorphous layer on the surface of the particles. In fact, a beneficial effect of Cr_2_O_3_ coatings of the cathode particles at high potential has been predicted by Wolverton and co-workers on the basis of extended density functional theory calculations^25^. Compared to the most recent literature available, the here proposed LNMO material outperforms all similar materials in terms of rate capability and extended cycling ability (see as an example the very recent review in ref. 11).

The voltage profiles of the TiO_2_-B electrodes half-cell, shown in Fig. 2c, highlight a very limited increase of the cell polarization upon increasing the current rate. The exchanged capacity ranges between 170 mAh g^−1^ at 100 mA g^−1^ to the value of ca. 125 mAh g^−1^ when moving to a 10 times higher current rate. The cycling stability of TiO_2_-nanotubes anode was investigated at different current-rates for over 1000 cycles. In the Fig. 2d the excellent performance stability of the TiO_2_-B electrode in a half cell configuration is illustrated: the exchanged specific capacity ranges from 150 mAh g^−1^ in the initial few cycles at 1000 mAg^−1^ to about 115 mAh g^−1^ after 1000 cycles at 100 mAg^−1^. The specific capacity decay during the first 100^th^ cycles of Fig. 2d can be ascribed to the electrolyte reaction at the surface of nano-tubes with formation of SEI layer. The main constituent of such SEI are Li_2_CO_3_, ROCO_2_Li_2_ (RO,F)_3_P = O and Li_x_PF_y_^22^. Furthermore, since the high-current rate used the TiO_2_-anode needs more than few cycles for the complete formation of the SEI-layer.

**Figure 3:**
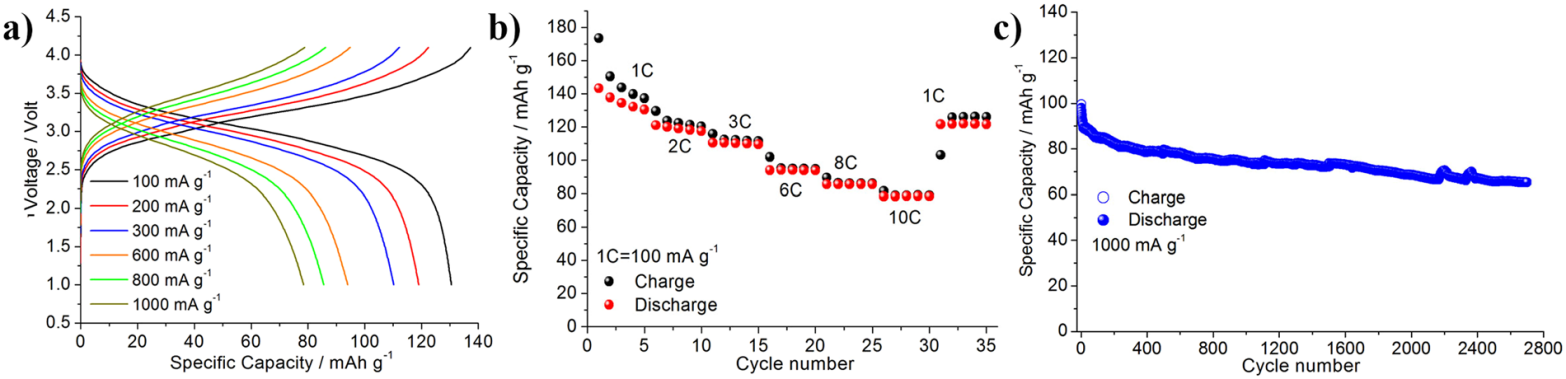
(**a**) Voltage profiles, (**b**) rate performance and (**c**) extended cycling-performance at 1000 mAg^−1^ of the TiO_2_-B/LP30/LNMO Li-ion cell.

Having established the performance of the negative and positive electrodes in half cells, a full cell configuration was assembled. All the Li-ion batteries here reported have been balanced by matching the cathode and anode capacity. The TiO_2_-B negative electrodes have not been electrochemically activated thank to the chemical mitigation of the irreversible capacity loss in the first cycle obtained by the chemical pre-treatment with lithium ethoxide (see the experimental section). For the sake of simplicity, the specific capacity values hereafter reported refer to the LNMO electrode mass. Figure 3 shows the rate capability and the prolonged galvanostatic response of the Li-ion cell cycled using current as high as 1000 mA g^−1^ (10C).

The voltage profiles shown in Fig. 3a reflect the combination of the voltage shape of the TiO_2_-B and of the LNMO electrodes. The analysis of the voltage profiles at different current rates reveals a specific capacity of 130 mAh g^−1^ at 100 mA g^−1^ (i.e. 1C), decreasing slightly to 80 mAh g^−1^ when the current is increased ten times (i.e. 1000 mA g^−1^, corresponding to 10C). The full-cell shows outstanding performance, comparable to that of the lithium half-cell (see and compare Fig. 2), with a capacity decreasing from 100 mAh g^−1^ to 80 mAh g^−1^ during the initial 500 cycles at 1000 mAg^−1^. Furthermore, between cycles 500 and 2700, the cell shows a remarkable cycling stability, with capacity only slightly decreasing. The final capacity retention at cycle 2700 exceed the 60% of the initial value at the 1^st^ cycle.

The mean working voltage of the cell is 2.85 V which results in an energy density of 230 Wh kg^−1^ at high current rate, i.e. 1000 mAg^−1^ (or 10C). Figure 3b gives a further demonstration of the excellent rate performance of this unique Li-ion configuration. In fact, the cell is able to cycle 60% of the initial specific capacity when the current rate is increased to 1000 from 100 mA g^−1^. Moreover, after 30 cycles performed at different current rates, 92% of the initial specific capacity is recovered when the specific current is lowered again to 100 mAg^−1^.

**Figure 4:**
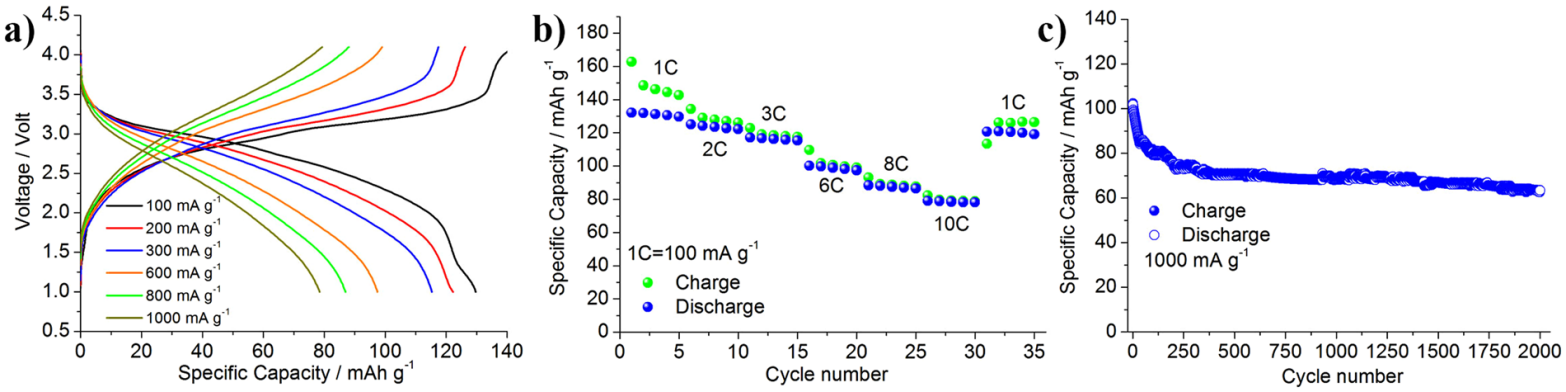
(**a**) Voltage profiles, (**b**) rate performance and (**c**) extended cycling performance at 1000 mAg^−1^ of the TiO_2_-B/LP30 + IL/LNMO Li-ion cell.

With the excellent performance of the full TiO_2_-B/LP30 /LNMO Li-ion battery using a standard carbonate based electrolytes, we now consider the final configuration with the incorporation of the composite electrolyte. Figure 4a shows the voltage profiles of the TiO_2_-B/LP30 + IL/LNMO cell. With the new electrolyte the hysteresis between charge and discharge increases at the highest current rates and the profiles become more and more sloping. However, the delivered capacity is almost unaltered. The current rate performance is also excellent, see Fig. 4b. Turning to the prolonged cycling performance shown in the Fig. 4, the cell is able to reversibly exchange 100 mAh g^−1^ during initial cycles at 1000 mAg^−1^ and retains approximately 65% of the initial capacity after 2000 cycles. The TiO_2_-B/LP30 + IL/LNMO cell operates at a working voltage of 2.85 V resulting in an energy density of 230 Wh kg^−1^ at high current rate, i.e. 1000 mAg^−1^ (10C), a value in line with the standard carbonate-based electrolyte cell (see Fig. 3c), but with the benefits of considerable safer electrolyte. The comparison between the galvanostatic performance of the cell using the pristine LP30 electrolyte and the one using the IL-added highlights minor differences in terms of electrochemical performances, e.g. slightly larger cell over-potentials, possibly attributed to minor alterations of the electrodes/electrolyte interface dynamics and consequent lower delivered specific capacity. A similar behavior between the full cells is given by the capacity decay during the first 100^th^ cycles. This can be ascribed to the phenomena occurring at the TiO_2_-anode side related with the SEI formation and further to the partial first irreversible capacity of the cathode material during the first charge. Further comparison reveals that the cell using the IL-added electrolyte has slightly higher Coulombic efficiency than the one using the pristine LP30 electrolyte, thus indicating that the addition of the IL in the LP30 solution is reducing the electrolyte reaction at high voltage. For better comparison and further discussion see Fig. S1 in the Supplementary Information section.

## Discussion

The successful coupling of an Fe- and Cr-doped LNMO spinel and TiO_2_-B nanotubes in Li-ion cells has been demonstrated: this formulation is able to sustain extended cycling and high current rates with minor capacity fade, thus disclosing outstanding performance in terms of energy and power capability, as well as in terms of long calendar life. Such excellent performance are confirmed both in a standard carbonate-based electrolyte and in an innovative composite electrolyte modified by the addition of a stable ionic liquid, aimed at the improvement of the overall safety of the battery. The LNMO electrode material here discussed differs from the state-of-art reported due to the unique synergic role played by the simultaneous iron doping and Cr-oxide coating. This composition tuning improves the structural stability, the electronic conductivity and prevents the electrolyte decomposition at high-voltage and C-rates. The final effect is the development of an electrode material having high cycling stability and excellent rate capability, prepared with an easy scalable and cheap synthetic route. On the negative electrode-side the adoption of TiO_2_-B nanotubes as active material improves the intrinsic safety of the device due to the working potential of the Ti^4+^/Ti^3+^ redox couple (i.e. above 1 V vs Li). Moreover, the unique morphology of this negative electrode material allows excellent rate performance and extended calendar life with minor capacity loss upon cycling. The combination of these two innovative electrode materials gives rise to a full Li-ion battery able to operate at 3 V, i.e. a viable voltage-range for energy storage applications, even at 10C-rate, by delivering an energy density as high as 230–240 Wh kg^−1^ and power densities higher than commercial and previously reported Li-ion systems (see Fig. S2 and related discussion in the Supplementary Information section). Furthermore, the Li-ion cell here developed showed an outstanding cycle-life, exceeding thousands cycles. With this work we marked a step forward in the field of future Li-ion battery, since the device here developed shows C-rates, energy performance and cycle-life never reported before.

## Methods

### Synthesis of LNMO and TiO_2_(B) powders material

Doped LiNi_0.5_Mn_1.5_O_4_ was prepared by a mixed mechano-chemical-solid state route. Oxides precursor were weighted and mixed by using a Spex 8000 M high energy miller for 5 hours in stainless-steal jars. The obtained powder was then heat-treated at 800 °C for 1 h under air with heating and cooling scan rates of 10 and 5 °C/min respectively.

Monoclinic TiO_2_(B) nanotubes were synthesized from anatase (Sigma Aldrich), by adding it to a solution of 15 mol/L NaOH (Sigma Aldrich) followed by hydrothermal treatment at 150 °C for 72 h^10^. Then the product of the hydrothermal reaction was washed with 0.05 mol/L of HCl (Sigma Aldrich), dried in air then heated to 400 °C for 5 h under O_2_-flow at 40 ml/min. After the annealing the synthesized TiO_2_(B) material has been transferred directly to the glove box without exposure in air. Before casting into film electrodes, the TiO_2_-B material has been treated with lithium ethoxide following the procedure illustrated by Brutti and co-workers in ref. 22 to mitigate the irreversible capacity loss in the first cycle. The preparation of the TiO_2_(B) based electrode films has been carried out in glove box to avoid the possible contamination with moisture.

### Materials characterization

Powder morphology was analyzed by means of a LEO 13 High Resolution Scanning Electron Microscope (SEM) and a FEI G2 20 HRTEM Transmission Electron Microscope (TEM).

### Electrodes preparation and electrochemical characterization

Electrode thin films were then prepared on Aluminum foils (Sigma Aldrich) by casting a dispersion of the active materials, 10% of Super P carbon (conducting agent, Timcal) and 10% PVdF (binder, Solef, 6020) in N-methyl pyrrolidone (NMP, Aldrich). The resulting 40 µm thick films were cut into disks and dried at 50 °C under vacuum to remove the residual solvent previous to lithium cell assembly. Electrodes were firstly tested in lithium metal cells, either using conventional LP30 SelectiLyte™ electrolyte (LiPF_6_ 1 M in an Ethylene Carbonate - Dimethyl Carbonate, 1:1 v:v solution) or the new electrolyte solution, the mixture of LP30 and 30 wt% of N-n-butyl-N-methylpyrrolidinium hexafluorophosphate (Py_14_PF_6_, Solvionic). Cells were realized in argon-filled glovebox (H_2_O and O_2_ content less than 1 ppm), using R2032 coin-type cells, 1.6 cm diameter, and Whatman glass fiber separators to support the electrolyte. Galvanostatic cycling tests at different current rates were carried out with a Maccor series 4000 battery tester, using voltage range between 5V–2.5 V and 1V–2.5 V for the LNMO and TiO_2_-nanotubes respectively. Therefore, TiO_2_-nanotubes/LP30 + Py_14_PF_6_/LNMO full cells were prepared by using the same cell configuration described above. The lithium-ion cell was assembled and properly balanced by a Positive/Negative capacity ratio of 1:1. The electrodes mass loading was about 2–3 mg/cm^2^. The Full Li-ion cell started in charge and cycled in the voltage range 4.1 V–1.0 V. No electrochemical pre-activation on both the electrodes was carried out before cycling.

## Electronic supplementary material

Supplementary Information
