# Supplementary material for: A high-power and fast charging Li-ion battery with outstanding cycle-life
Source: Sci Rep. 2017 Apr 24;7:1104. doi: 10.1038/s41598-017-01236-y (PMC5430621; doi:10.1038/s41598-017-01236-y)
Supplement: Supplementary file 1 — Supplementary Information [file 41598_2017_1236_MOESM1_ESM.pdf]

A high-power and fast charging Li-ion battery with outstanding cycle-life

M. Agostini*[1,4], S. Brutti[2,3], M.A. Navarra[4], S. Panero[4], P. Reale[5], A. Matic[1], B. Scrosati*[6]

[1]Department of Applied Physics, Chalmers University of Technology SE-41296 Göteborg, Sweden
[2]CNR-ISC, U.O.S. Sapienza, Piazzale A. Moro 5, 00185 Roma, Italia
[3]Dipartimento di Scienze, Università della Basilicata, V.le Ateneo Lucano 10, 85100 Potenza, Italia
[4]Dipartimento di Chimica, Sapienza Università di Roma, P.le Aldo Moro 5, 00185 Roma, Italia
[5]ENEA-Centro di Ricerca Casaccia, Via Anguillarese, 00100, Roma, Italia
[6] Helmholtz-Institut Ulm (HIU), Ulm, Germany
*corresponding authors: bruno.scrosati@gmail.com; agostini@chalmers.se

SUPPLEMENTARY INFORMATION

Figure S1 reports the comparison between the galvanostatic performance of the cell using the LP30 electrolyte (blue dots and line) and the one using the IL-added electrolyte (red dots and line). Main changes are given by differences in cell over-potentials, possibly attributed to minor alterations in the electrodes/electrolyte interfaces and consequently the lower delivered specific capacity. Furthermore, the cell using the IL-added electrolyte shows higher Coulombic efficiency then the one using the pristine LP30 solution thus confirming a reduction in the electrolyte decomposition at the higher voltage.

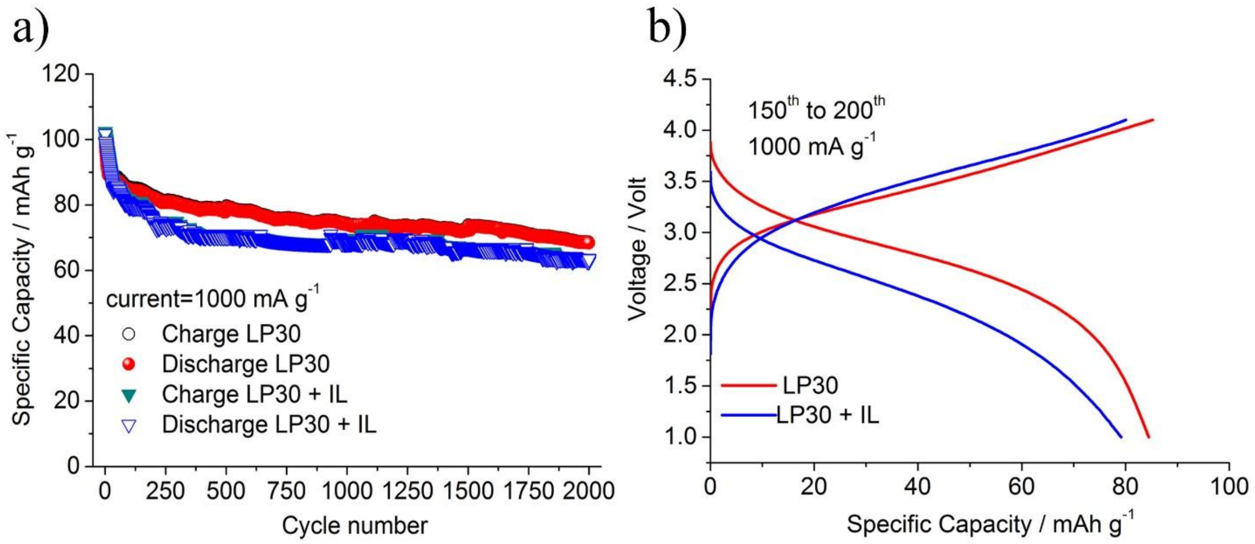

Figure S1. Comparison between the (a) prolonged cycling performance and (b) the voltage profiles Li-ion cells using the pristine LP30 electrolyte (red) and the IL-added electrolyte (blue). Current rate of 1000 mA g^{-1}.

Figure S2 reports the ragon plot for the Li-ion cell using the IL-added electrolyte, the TiO_2-nanotubes and the LNMO cathode. The practical energy densities and the power densities have been calculated taking into account the weight of cathode, anode and electrolyte. The first component is considered as the active part for the calculation of the theoretical energy and power while the other two components are only considered for the calculation of the practical values. The Figure shows energy and power densities values comparable to those of commercial Li-ion systems at the lower current-rates. Main differences are evidenced at the higher current-rate where the Li-ion systems here proposed shows practical power and energy densities higher than commercial and future Li-ion systems[1-5]. Furthermore, comparing to other systems reported in the state of art, the device here proposed showed outstanding cycling duration[4,5].

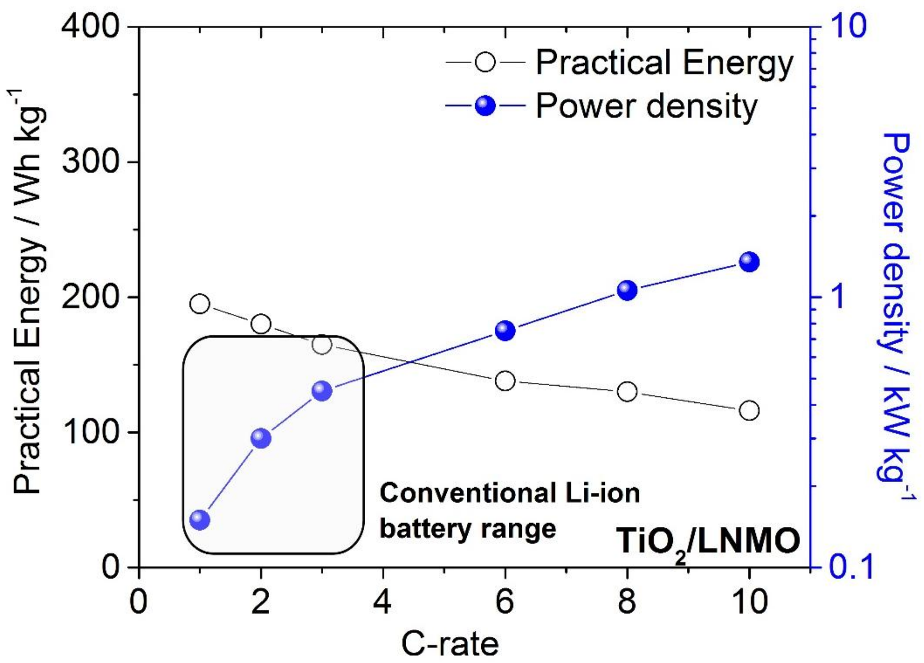

Figure S2. Practical energy (Wh kg^{-1}) and power density (kW kg^{-1}) at different current rate (1C=100 mA g^{-1}) calculated for the Li-ion cell using the IL-LP30 added electrolyte, the TiO_2-nanotubes anode and the LNMO cathode. The figure is also evidencing the range corresponding to the conventional Li-ion batteries.

References

[1] Agostini, M., Brutti, S. & Hassoun, J. High Voltage Li-Ion Battery Using Exfoliated Graphite/Graphene Nanosheets Anode. *ACS Appl. Mater. Interfaces*. **8**, 10850-10857, (2016).

[2] Chung, S.Y., Bloking, J.T. & Chiang, Y.M. Electronically conductive phospho-olivines as lithium storage electrodes. *Nat. Mater*. **1**, 123-128, (2002).

[3] Winter, M., Besenhard, J.O., Spahr, M.E. & Novak, P. Insertion Electrode Materials for Rechargeable Lithium Batteries. *Adv. Mater.* **10**, 725-763, (1998).

[4] Jung, H.G., Jang, M.W., Hassoun, J., Sun, Y.-K. & Scrosati, B. A high-rate long-life Li4Ti5O12/Li[Ni0.45Co0.1Mn1.45]O4 lithium-ion battery. *Nat. Commun.* **2**, 516, (2011).

[5] Elia, G., Ulissi, U., Jeong, S., Passerini, S. & Hassoun, J. Exceptional long-life performance of lithium-ion batteries using ionic liquid-based electrolytes. *Energy Environ. Sci.* **9**, 3210-3220, (2016).
